# Epicuticular Wax Composition and Wax Crystal Microstructure Shape Oviposition Preference in the Diamondback Moth, *Plutella xylostella*, on Cabbage

**DOI:** 10.1007/s10886-026-01778-4

**Published:** 2026-11-03

**Authors:** Kodai Inoue, Manaka Hata, Hisashi Ômura

**Affiliations:** 1 Graduate School of Integrated Sciences for Life, Hiroshima University, Higashihiroshima, 739-8528 Japan; 2 School of Applied Biological Science, Hiroshima University, Higashihiroshima, 739-8528 Japan; 3 Seto Inland Sea Carbon-neutral Research Center, Hiroshima University, Higashihiroshima, 739-8528 Japan

**Keywords:** Diamondback moth, Cabbage leaf, Epicuticular wax, Oviposition preference, Glossy and waxy cultivars, Long-chain alkane

## Abstract

The diamondback moth *Plutella xylostella* is a major pest of cabbage and exhibits distinct host preferences among cabbage cultivars. Glossy cabbage cultivars, characterized by reduced epicuticular wax crystal coverage on the leaf surface, are generally more susceptible to oviposition by females than waxy cultivars with normal wax bloom. However, the roles of wax components in shaping oviposition behavior remain unclear. We examined this issue using the waxy cultivar ‘Kinkei 201 (Kinkei)’ and the glossy cultivar ‘YR Yuryoku (YR)’. In two-choice tests, females significantly preferred YR over Kinkei for oviposition. Kinkei leaves were densely covered with well-developed epicuticular wax crystals dominated by *n*-nonacosane (nC29), whereas YR leaves had sparse, granule-like wax crystals dominated by triacontanal (C30al). Mechanical removal of wax crystals significantly increased oviposition on Kinkei leaves but not on YR leaves. Application of 0.1–10 mg nC29 to wax-removed Kinkei leaves significantly suppressed oviposition and, at higher doses, induced dense wax structures across the leaf surface. Similarly, application of 1 mg nC29 to intact YR leaf surfaces increased nC29 coverage and significantly reduced oviposition. In contrast, application of C30al to wax-removed YR leaf surfaces produced sparse wax structures and did not affect oviposition. These results suggest that oviposition deterrence is associated with dense nC29-associated wax structures rather than simply with the presence of epicuticular wax itself. Differences in oviposition preference between the two cabbage cultivars may reflect variation in both epicuticular wax composition and wax crystal microstructure, although their relative contributions remain unclear.

**Supplementary Information:**

The online version contains supplementary material available at https://doi.org/10.1007/s10886-026-01778-4.

## Introduction

The diamondback moth, *Plutella xylostella* (L.) (Lepidoptera: Plutellidae), is a specialist herbivore of Brassicaceae plants that has evolved adaptations to the chemical defenses of its hosts based on glucosinolates (GLSs) (Ratzka et al. 2002; Textor and Gershenzon 2009). It is one of the most destructive pests of *Brassica* crops worldwide, causing severe yield losses (Farias et al. 2020) and generating annual management costs estimated at US$4–5 billion (Zalucki et al. 2012). Control of this pest has relied largely on synthetic insecticides, but the widespread evolution of insecticide resistance has increased the need for alternative management strategies (Banazeer et al. 2022; Sarfraz and Keddie 2005). Therefore, a better understanding of plant traits that influence host selection and performance could facilitate the development of Brassicaceae cultivars with enhanced resistance to *P*. *xylostella* (Mitchell et al. 2016; Sarfraz et al. 2007; Stout 2013).

Since lepidopteran larvae often settle on the plants where they hatch, host selection by female adults during oviposition is crucial for offspring survival and development (Renwick and Chew 1994). Consistent with this expectation, *P*. *xylostella* generally exhibits a positive correlation between adult oviposition preference and larval performance (Fathi et al. 2011; Zhang et al. 2012). Like other Brassicaceae-feeding specialists, female diamondback moths use plant-derived chemical cues to locate and assess host plants. Volatile compounds emitted from host plants mediate long-range host location, whereas non-volatile secondary metabolites encountered after landing influence host acceptance and oviposition decisions (Badenes-Pérez et al. 2004; Couty et al. 2006; Justus and Mitchell 1996). Numerous studies have identified specific volatile compounds, GLSs, and GLS-derived products that either stimulate or deter oviposition in *P*. *xylostella* (Gupta and Thorsteinson 1960; Renwick et al. 2006; Sun et al. 2009). Thus, the chemical basis of host selection by female diamondback moths is relatively well understood with respect to plant volatiles and secondary metabolites.

In addition to these compounds, leaf epicuticular wax is known to affect oviposition behavior in *P. xylostella*. Epicuticular wax forms the outermost interface between insects and plant surfaces and consists of a complex mixture of long-chain aliphatic compounds arranged as wax films and crystals (Müller and Riederer 2005; Žnidarčič et al. 2008). In Brassicaceae crops, females generally prefer cultivars with reduced epicuticular wax crystal coverage, referred to as glossy cultivars, over waxy cultivars with normal wax bloom (Justus et al. 2000; Ulmer et al. 2002). Likewise, artificial removal of epicuticular wax from host plant leaves often increases oviposition (Silva et al. 2017; Zhu et al. 2022). Interestingly, despite being more attractive for oviposition, glossy cultivars are often less susceptible to larval infestation than waxy cultivars under field conditions (Eigenbrode et al. 1991; Stoner 1990). These findings suggest that epicuticular wax may influence female oviposition and larval performance through different mechanisms. However, unlike volatile compounds and secondary metabolites, it remains unclear which epicuticular wax components and associated wax structures influence oviposition behavior in *P. xylostella*.

This study aimed to determine whether major wax components characteristic of waxy and glossy cabbage cultivars contribute to oviposition preference in the diamondback moth and to evaluate the effects of different application levels on oviposition behavior. First, we conducted chemical analyses to determine the epicuticular wax composition of the two cultivars. Second, under laboratory conditions, we performed oviposition bioassays using females that had not previously laid eggs. In the bioassays, we examined oviposition preferences of females between cabbage leaf surfaces with different wax profiles: (1) intact leaves of a waxy cultivar versus those of a glossy cultivar; (2) paired wax-removed surfaces, one treated with an authentic wax component and the other serving as a control; and (3) paired intact surfaces, one treated with an authentic wax component and the other serving as a control. Third, we characterized the microstructure of leaf surfaces used in the bioassays and examined its potential association with oviposition preference.

## Materials and Methods

### Plants

Two cabbage (*B*. *oleracea* var. *capitata*) cultivars–a waxy cultivar, ‘Kinkei 201’ (Kinkei: Sakata Seed Co., Yokohama, Japan), and a glossy cultivar, ‘YR Yuryoku’ (YR: Marutane Co., Kyoto, Japan)–were used in this study. Planting soil was prepared by mixing potting soil and sandy soil in a 1:1 volume ratio, sieving through an 8.6 mesh size (2 mm), and air-drying. Two cabbage seeds were sown in plastic pot (8.5 cm height, 8.3 cm upper inner diameter) containing approximately 200 mL planting soil and maintained under controlled conditions of temperature 20 ± 1 °C and photoperiod 16 L:8D. The potted seedlings were irrigated to maintain soil moisture content at approximately 60%. Two weeks after sowing, the two seedlings were thinned to one per pot. The seedlings with 5–7 leaves, 6–7 weeks after sowing, were used for chemical analyses and bioassays.

### Insects

The insecticide-susceptible strain of *P*. *xylostella* was provided by the National Agriculture and Food Research Organization (NARO) Genebank, Tsukuba city, Ibaraki Prefecture, Japan. Insects were subcultured using a modified method of Toyoshima and Kobayashi (2001) and maintained under conditions of temperature 20–23 °C and photoperiod 16 L:8D. Mated females were allowed to lay eggs on *Brassica* seedlings in 5 mL vials. Larvae were reared on radish sprouts in transparent plastic vessels (9 cm height, 8 cm inner diameter). Adults were transferred into transparent plastic cups without host plants and fed 10% sucrose solution. Females observed in copula were collected and maintained individually until use. Because no oviposition substrate was provided before the bioassays, females had no opportunity to lay eggs prior to the experiments. Bioassays were conducted using these mated, oviposition-naïve females 3 days after emergence.

### Cabbage Leaf Wax Extraction

Second to fourth leaves from the top of 7-week-old cabbage seedlings were detached at the petiole base. Each leaf was immersed in 40 mL of chloroform for 30 s to extract epicuticular wax. From this solution, a 10 mL portion was transferred to a screw-capped vial and stored at -20 °C. Immediately after extraction, each leaf was air-dried, placed between transparent glass plates, and photographed by digital camera to determine the leaf area using Image J 1.54d image analysis software. For each cabbage cultivar, ten samples were prepared using different seedlings.

### Chemical Analysis

The cabbage leaf wax extracts were subjected to gas chromatography-electron ionization mass spectrometry (GC-EIMS), using GC-17A gas chromatography (Shimadzu, Kyoto, Japan) equipped with a Supelco Equity-1 capillary column (15 m × 0.25 mm ID, 0.25 μm film thickness: Bellefonte, PA, USA) and QP5000 mass spectrometer (Shimadzu, Kyoto, Japan) at an EI voltage of 70 eV. A 2 mL portion of the stocked sample was concentrated to 100 µL under a gentle nitrogen stream at 60 °C. One µL of the concentrated sample was injected into the capillary column in splitless mode with an injector temperature of 280 °C and a split opening 30 s after injection. The temperature of column oven was programmed from 50 °C (initial 2 min hold) to 280 °C (final 10 min hold) at 10 °C/min. The wax components were identified by comparing their retention times and mass spectra with those of chemical standards or were tentatively identified based on published data (Holloway and Brown 1977; Netting and Macey 1971). *n*-Nonacosane (nC29) in each sample was quantified with a calibration curve of its authentic chemical, while other components were semi-quantified by comparing their peak areas with that of nC29. A calibration curve for nC29 was generated using 0.1 and 1.0 µg of the authentic compound (*y* = 290.7*x* + 1.473, *R*² = 1.00, where *y* is peak area and *x* is the amount of nC29). Extraction recovery, injection variability, and analyte loss during sample preparation were not formally evaluated. The content of each component per unit leaf area was then calculated.

### Chemical Standards

nC29 (purity > 98%) was purchased from Tokyo Chemical Industry (Tokyo, Japan). Triacontanal (C30al: purity > 90%) was synthesized via conventional pyridinium chlorochromate oxidation from authentic 1-triacontanol (purity > 85%) supplied by Tokyo Chemical Industry and purified by silica gel column chromatography. To prevent oxidation, synthesized C30al was dissolved in chloroform and stored at -20 °C.

### Foliar Treatments

To modify the properties of leaf epicuticular wax, second to fourth leaves from the top of cabbage seedlings older than 7 weeks were detached at the base of the petiole. For each detached leaf, either the left or right half relative to the midvein was randomly selected, and both the adaxial and abaxial surfaces were subjected to the following treatments.

1. Mechanical wax removal: Leaf epicuticular wax crystals were gently removed by rubbing the surface with clean cotton balls to avoid scratching. One half of each test leaf was subjected to the wax removal treatment, and the other half was left untreated as a control. In Kinkei, this treatment reduced nC29 content on the leaf surface to 37% of that in the control (Ueno et al. 2025). In YR, 80% of nC29 and 90% of C30al remained on the leaf surface after this treatment (Online Resource 1).
2. Spraying with chemical standards: Using a hobby airbrush (0.3 mm nozzle size, 25 psi maximum air pressure), nC29 and C30al standards were sprayed onto the surface of the test leaves. The nC29 standard (0.1, 1, or 10 mg) was dissolved in 5 mL of isopentane, while a chloroform solution (< 300 µL) containing 1 mg of C30al was diluted with the same solvent. These solutions were loaded into the airbrush and sprayed onto both the adaxial and abaxial surfaces of selected half of each leaf. The other half was covered with aluminum foil to prevent application of the test chemicals.
  After spraying, the treated half was completely air-dried and then covered with aluminum foil. Subsequently, the other half was uncovered, sprayed with 5 mL of isopentane alone, and allowed to air-dry thoroughly. The aluminum foil covering the treated half was then removed.
  When nC29 solutions (0.1, 1, and 10 mg) were sprayed onto the wax-removed halves of Kinkei leaves, the amounts of nC29 deposited on the treated surface were estimated to be 7, 56, and 360 µg/cm^2^, respectively, based on values reported by Ueno et al. (2025). These deposition levels were not directly measured in the present study. Spraying a solution containing 1 mg of C30al onto the wax-removed halves of YR leaves approximately doubled the C30al content, increasing it from 5.5 µg/cm^2^ on the wax-removed surface to 12.3 µg/cm^2^ on the treated surface (Online Resource 2).
  When nC29 solutions (0.1 and 1 mg) were sprayed onto the intact halves of YR leaves containing 3.3–3.6 µg/cm^2^ of nC29, the amounts of nC29 deposited on the treated surface were estimated to be 5.0 and 18.0 µg/cm^2^, respectively (Online Resource 3). Application of 5 mL of solvent to intact Kinkei and YR leaf surfaces did not significantly affect oviposition by *P*. *xylostella* (Online Resource 4).

### Oviposition Bioassay

Two-choice tests were performed to examine the effects of cabbage leaf epicuticular wax on the oviposition preference of *P*. *xylostella*. Eight mated females were randomly selected from the stock culture to form one group. Each group was transferred into a transparent box-shaped chamber (34 cm × 26 cm × 34 cm), and subjected to the following experiments: (1) an intact leaf of Kinkei versus an intact leaf of YR, (2) the intact half versus the wax-removed half of a single leaf of Kinkei or YR, (3) the solvent-sprayed half versus the nC29-treated half of a single wax-removed leaf of Kinkei, (4) the solvent-sprayed half versus the C30al-treated half of a single wax-removed leaf of YR, and (5) the solvent-sprayed half versus the nC29-treated half of a single intact leaf of Kinkei or YR. nC29 was applied at 0.1, 1, or 10 mg per leaf, whereas C30al was applied at 1 mg per leaf. Each test leaf was placed vertically in a 5 mL glass vial (8 mm ID top, 45 mm height) containing water, and the vial was placed at the center of the chamber floor. In experiment (1), two vials, each containing one leaf of Kinkei or YR, were placed 10 cm apart at the center.

The bioassays were conducted in the dark at a laboratory room temperature of 25 °C. In the test chamber, eight females were allowed to fly and oviposit freely for 3 h. To compensate for positional effects between treated and control leaf surfaces, the leaf orientation was reversed 1.5 h after the start of bioassay. Females of *P*. *xylostella* are known to preferentially lay eggs on the concavities, dimples, and grooves of leaves, stems, and artificial substrates (Gupta and Thorsteinson 1960; Justus and Mitchell 1996; Uematsu and Sakanoshita 1993). Preliminary observations showed that females frequently rested upside down on the threaded portion of the vial opening and deposited eggs on adjacent leaf surfaces that could be reached by the abdomen. As a result, eggs were often concentrated on leaf areas located within 1 cm of the vial opening, irrespective of leaf wax properties (Online Resource 4). Therefore, to minimize potential positional effects associated with the vial structure, we counted only eggs deposited on leaf areas located more than 1 cm from the vial opening. Eggs deposited on the vial itself or within 1 cm of the vial opening were excluded. Egg counts from the adaxial and abaxial surfaces were pooled for all subsequent analyses. Each type of experiments was repeated 10–20 times using different groups of females.

### Data Analysis

Data analyses were conducted using R software version 4.3.1 (R Development Core Team 2023). In chemical analyses, significant differences in amount of each component between Kinkei and YR were analyzed with the Mann-Whitney *U* test using the package ‘wilcox.test.’ In oviposition bioassays, significant differences in female choice between the two leaf surfaces with different wax properties were analyzed with the quasibinomial generalized linear model (GLM). Furthermore, the oviposition proportion between the two leaf surfaces was calculated from the number of eggs laid in each trial and averaged across trials.

### Leaf Surface Morphology

Scanning electron microscopy (SEM) was performed to investigate the surface microstructure of the cabbage leaves used in the bioassays. True leaves detached from seedlings of Kinkei and YR were used intact or after implementation of either of the foliar treatment described above. A test piece (5 mm × 5 mm) was cut from each leaf sample and attached to an SEM cylinder mount using double-sided tape. The leaf piece on the mount was rapidly frozen using an FDC10 cooling unit (SUN Technologies, Tokyo, Japan) at -100 °C, dehydrated using an FD-6510 freeze dryer (SUN Technologies, Tokyo, Japan), and the sputter-coated with Pt using a JFC-1600 auto fine coater (JEOL, Akishima, Japan). The leaf surface morphology was examined using a JSM-5610 LV SEM (JEOL, Akishima, Japan) or a JSM-5400 SEM (JEOL, Akishima, Japan) at 10 or 15 kV, with images acquired at a magnification of 2,000×. For each condition, including the intact (untreated) control, two leaves were analyzed. SEM observations were conducted at five locations on a tissue piece excised from each leaf, yielding a total of 10 observation sites per condition.

## Results

### Oviposition Preference Between Kinkei and YR

**Fig. 1:**
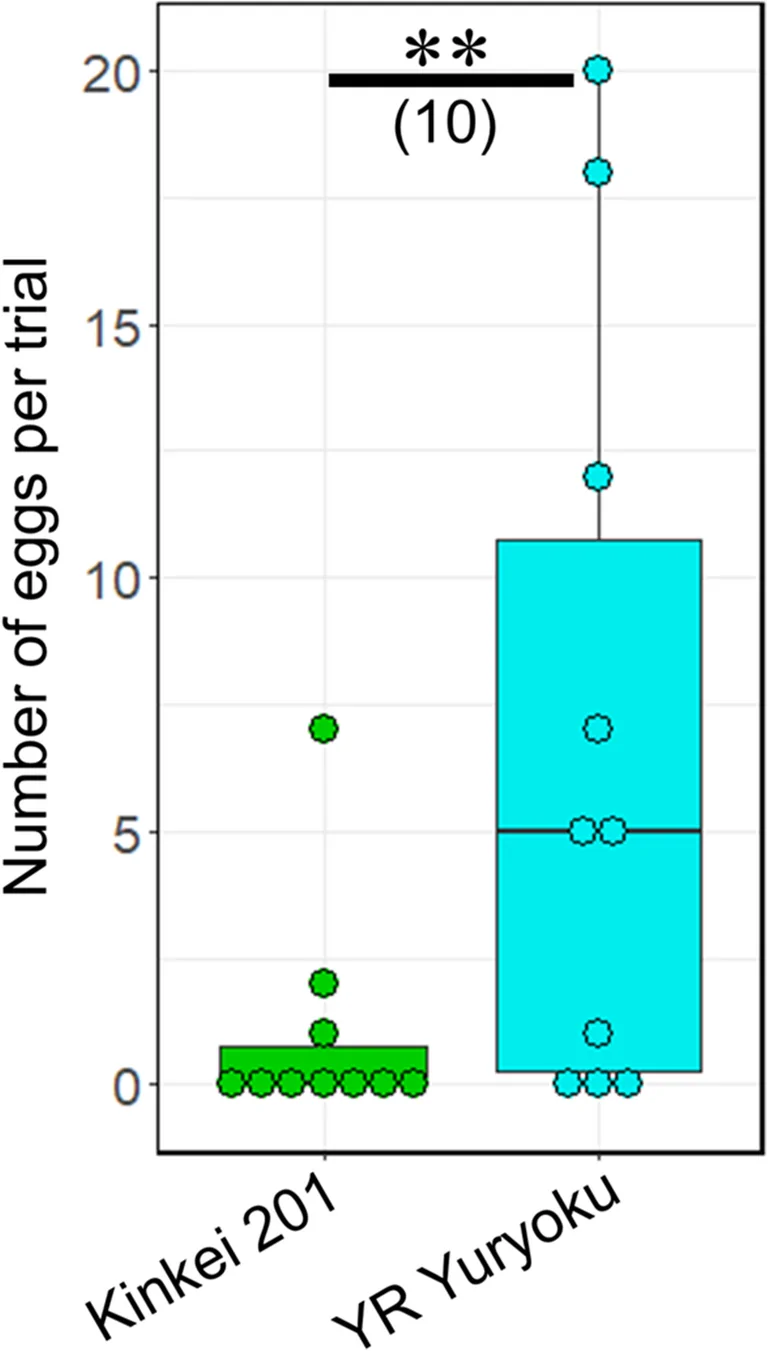
Oviposition preference of *Plutella xylostella* females between an intact leaf of cabbage cultivar Kinkei 201 and that of cultivar YR Yuryoku. The number of trials is shown in parentheses. Asterisks indicate statistically significant differences (Quasibinomial GLM: ** for *P* < 0.01)

When exposed to intact leaves of Kinkei and YR simultaneously, *P*. *xylostella* females laid significantly more eggs on that of YR (Fig. 1, quasibinomial GLM: *P* = 0.00803, *n* = 10). The mean oviposition proportion on YR was 0.896.

### Effects of Wax Removal on Female Oviposition

**Fig. 2:**
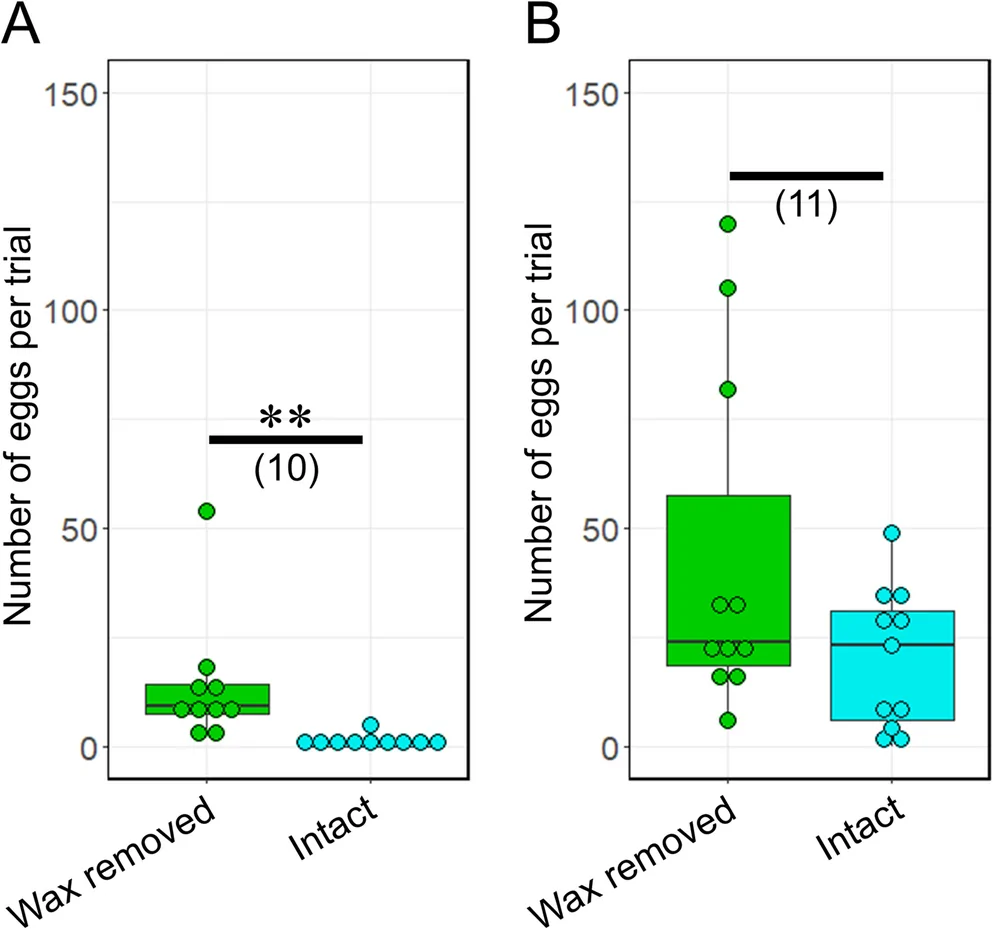
Oviposition preference of *Plutella xylostella* females on a single leaf of cabbage cultivars (**a**) Kinkei 201 and (**b**) YR Yuryoku, with different treatments applied onto the left and right halves separated from the midvein. Wax-removed half versus intact half with natural epicuticular wax. Numbers in parentheses denote the number of trials. Asterisks indicate statistically significant differences (Quasibinomial GLM: ** for *P* < 0.01)

Mechanical wax removal increased female oviposition on the treated surfaces of cabbage leaves (Fig. 2). In Kinkei, females showed a strong preference for the wax-removed surface, laying an average of 95.5% of their eggs on the treated surface (Fig. 2a, quasibinomial GLM: *P* = 0.0016, *n* = 10). In YR, females also laid more eggs on the wax-removed surface than on the intact surface, with a mean oviposition proportion of 0.633 on the treated surface; however, this difference was not significant (Fig. 2b, quasibinomial GLM: *P* = 0.0636, *n* = 11).

### Difference in Chemical Profile of Leaf Epicuticular Wax

The two cabbage cultivars, Kinkei and YR, shared four long-chain aliphatic compounds in their leaf epicuticular waxes, but their chemical compositions were significantly different (Table 1, Supplementary Fig. S1). The predominant component was nC29 in Kinkei, whereas it was C30al in YR. Furthermore, 15-nonacosanone and 15-nonacosanol were tentatively identified as minor components based on published data (Holloway and Brown 1977; Netting and Macey 1971). Kinkei had significantly greater amounts of nC29, 15-nonacosanone, and 15-nonacosanol than YR (Mann-Whitney *U* test: W = 100, *P* < 0.001), whereas YR contained significantly greater amounts of C30al than Kinkei (Mann-Whitney *U* test: W = 4, *P* < 0.001). Furthermore, the total amounts of the four components per unit leaf area were estimated to be 16.1 µg/cm² for Kinkei and 6.8 µg/cm² for YR respectively, indicating that Kinkei contained approximately 2.4 times more epicuticular wax than YR.

**Table 1:** Composition of leaf surface wax from 7-week-old seedlings of cabbage cv. Kinkei 201 and cv. YR Yuryoku

No.	Retention time (min)^a^	Compound	Amount^b^ (µg/cm^2^; mean ± SD, *n* = 10)		
			Kinkei 201	YR Yuryoku	Ratio (Kinkei 201 : YR Yuryoku)
1	22.98	*n*-Nonacosane	7.61 ± 0.43***^c^	1.22 ± 0.15	6.2 : 1
2	24.06	15-Nonacosanone^d^	4.22 ± 1.00***	1.12 ± 0.21	3.8 : 1
3	24.21	15-Nonacosanol^d^	2.35 ± 0.73***	0.12 ± 0.04	19.6 : 1
4	25.07	Triacontanal	1.91 ± 0.99	4.29 ± 0.27***	1 : 2.2

^a^On a Supelco Equity-1 capillary column (15 m × 0.25 mm ID), programmed from 50 °C (initial 2 min hold) to 280 °C (final 10 min hold) at a temperature increase of 10 °C/min

^b^*n*-Nonacosane was quantified with an external calibration curve of the authentic chemical, whereas all other compounds were semi-quantified relative to *n-*nonacosane based on peak areas

^c^Significant differences between Kinkei 201 and YR Yuryoku (Mann-Whitney *U* test: *** for *P* < 0.001)

^d^Tentative identification based on published data (Holloway and Brown 1977; Netting and Macey 1971)

### Effects of Foliar Application of nC29 on Female Oviposition in Kinkei

**Fig. 3:**
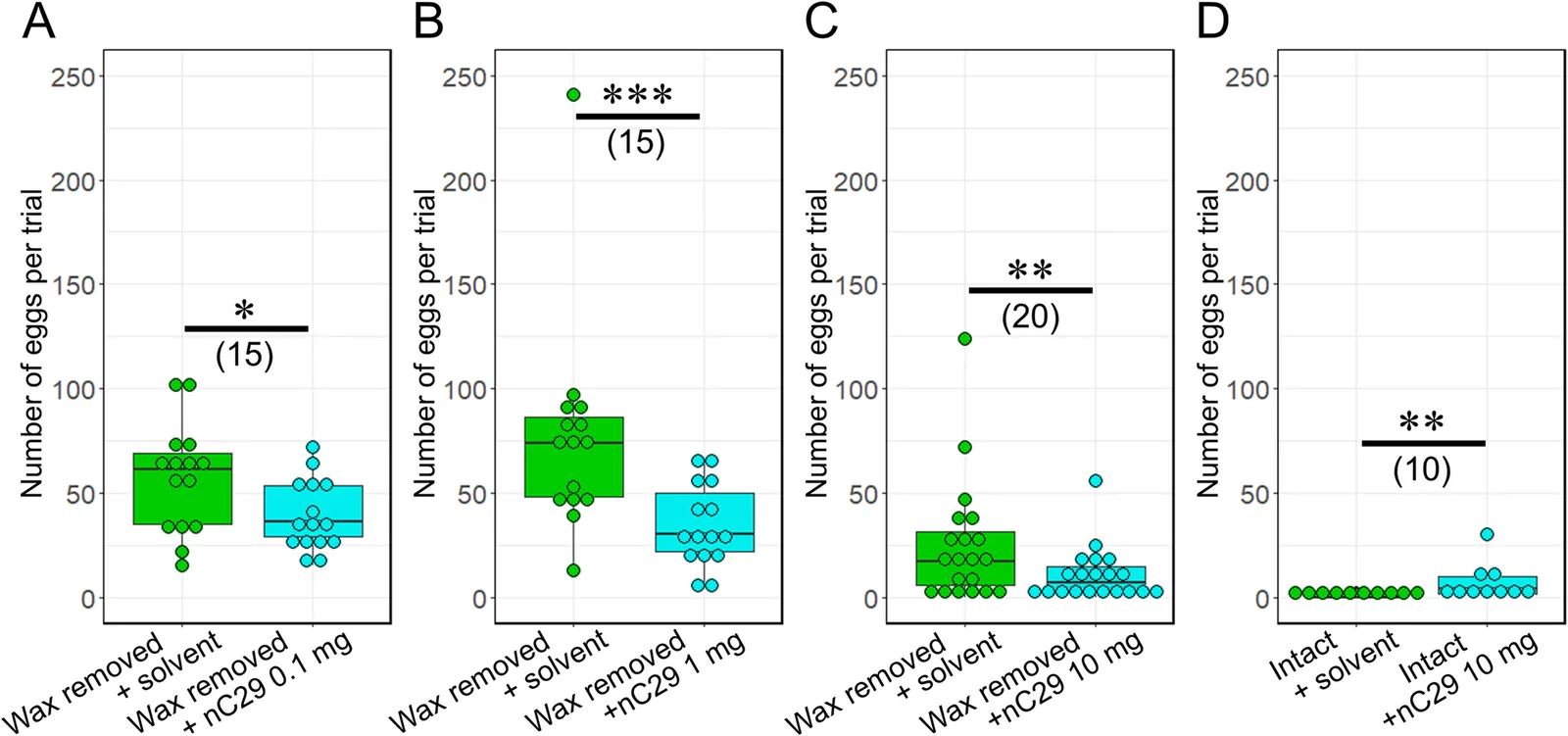
Oviposition preference of *Plutella xylostella* females on a single leaf of cabbage cultivar Kinkei 201, with different treatments applied onto the left and right halves separated from the midvein. **a**-**c** Half with wax-removed and sprayed with isopentane solvent versus half with wax-removed and sprayed with solutions containing 0.1 mg, 1 mg, or 10 mg of *n*-nonacosane (nC29). **d** Half sprayed with isopentane solvent versus half sprayed with an isopentane solution containing 10 mg of nC29. Numbers in parentheses denote the number of trials. Asterisks indicate statistically significant differences (Quasibinomial GLM: * for *P* < 0.05, ** for *P* < 0.01, and *** for *P* < 0.001)

Using Kinkei leaves, we examined the effect of nC29 application on the oviposition of *P*. *xylostella*. Spraying 0.1, 1, or 10 mg of nC29 onto the wax-removed surface significantly reduced female oviposition on the treated surface (Fig. 3a–c, quasibinomial GLM: *P* = 0.0227, 0.0001, and 0.0039 for 0.1-, 1-, and 10-mg treatments, respectively). The mean oviposition proportions on the treated surfaces were 0.423 (*n* = 15), 0.428 (*n* = 15), and 0.316 (*n* = 20) for the 0.1-, 1-, and 10-mg treatments, respectively. The mean total numbers of eggs deposited (on both control and treated surfaces) per trial were 97, 111, and 36 in the 0.1-, 1-, and 10-mg treatments, respectively. In contrast, spraying 10 mg of nC29 onto the intact surface significantly increased female oviposition on the treated surface, with a mean oviposition proportion of 0.912 and a mean total egg deposition of 8 per trial (Fig. 3d, quasibinomial GLM: *P* = 0.0068, *n* = 10).

### Effects of Foliar Application of nC29 and C30al on Female Oviposition in YR

**Fig. 4:**
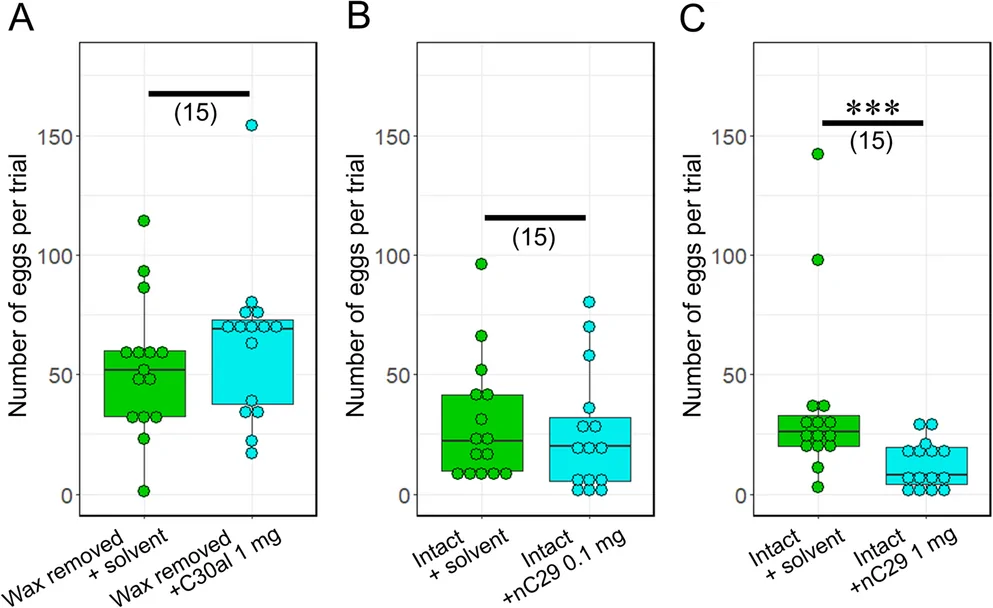
Oviposition preference of *Plutella xylostella* females on a single leaf of cabbage cultivar YR Yuryoku, with different treatments applied onto the left and right halves separated from the midvein. **a** Half with wax-removed and sprayed with a mixed solvent (isopentane containing a small amount of chloroform) versus half with wax-removed and sprayed with a solution containing 1 mg of triacontanal (C30al). **b**-**c** Half sprayed with isopentane solvent versus half sprayed with solutions containing 0.1 mg or 1 mg of *n*-nonacosane (nC29). Numbers in parentheses denote the number of trials. Asterisks indicate statistically significant differences (Quasibinomial GLM: *** for *P* < 0.001)

In YR, spraying 1 mg of C30al onto the wax-removed surface slightly increased female oviposition (mean oviposition proportion on the C30al-treated surface = 0.550; mean total number of eggs per trial = 116); however, the difference was not significant (Fig. 4a, quasibinomial GLM: *P* = 0.392, *n* = 15). Application of 0.1 mg of nC29 to the intact surface of YR slightly reduced oviposition, although the reduction was not significant (Fig. 4b, quasibinomial GLM: *P* = 0.47, *n* = 15; mean oviposition proportion on the nC29-treated surface: 0.414). In contrast, spraying 1 mg of nC29 onto the intact surface significantly suppressed female oviposition on the treated surface (Fig. 4c, quasibinomial GLM: *P* < 0.0001, *n* = 15; mean oviposition proportion on the nC29-treated surface: 0.267). The mean total numbers of eggs deposited per trial were 55 and 49 in the 0.1- and 1-mg treatments of nC29, respectively.

### Differences in Leaf Surface Microstructures

**Fig. 5:**
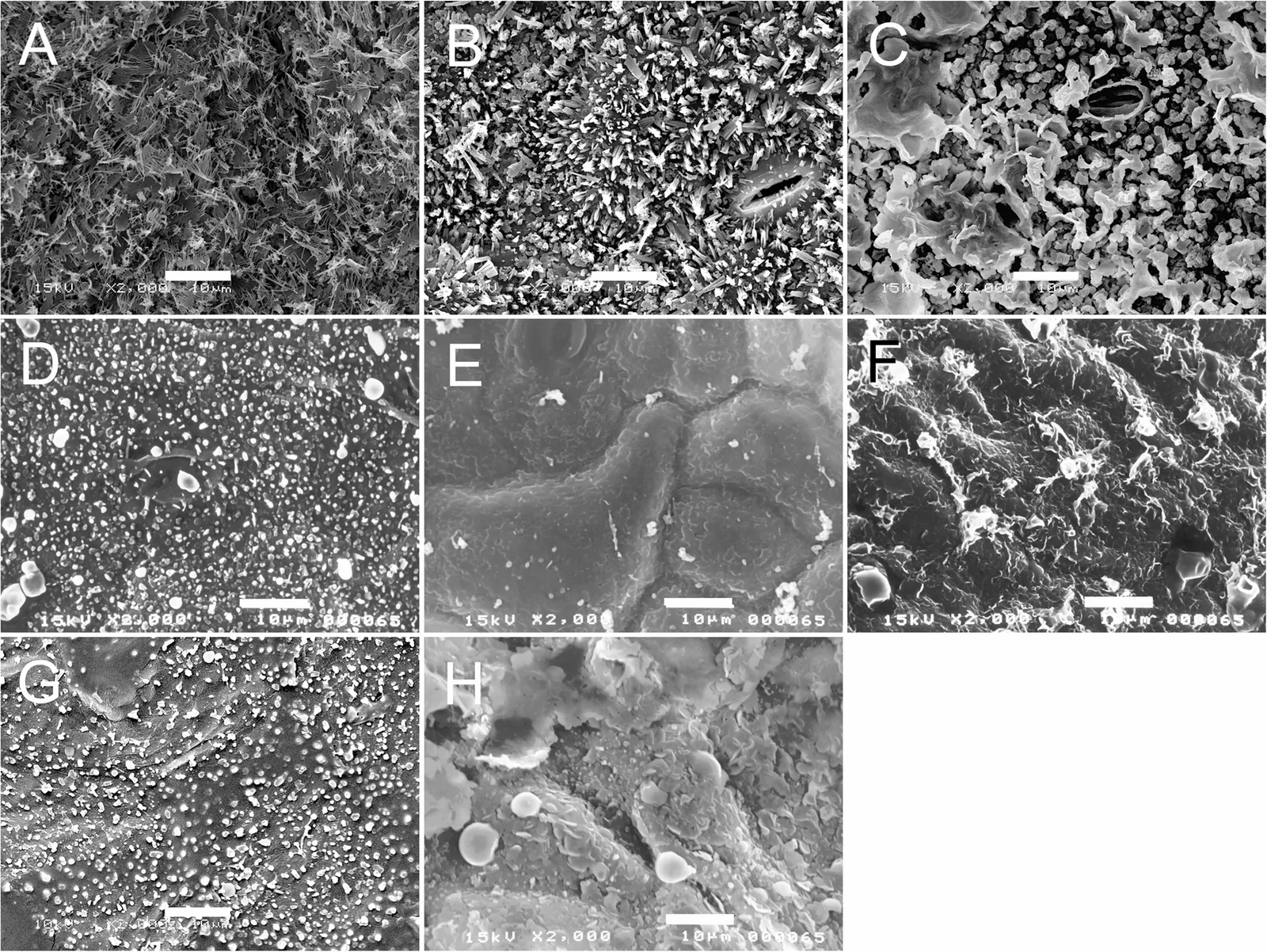
Scanning electron micrographs of the abaxial surfaces of true leaves of cabbage cultivars Kinkei 201 (**a**-**b**) and YR Yuryoku (**d**-**h**) used in the bioassays. **a** No treatment (intact). **b** Sprayed with 5 mL of isopentane. **c** Sprayed with 5 mL of an isopentane solution containing 10 mg of *n*-nonacosane. **d** No treatment (intact). **e** Wax removal by rubbing with cotton balls. **f** Wax removal and sprayed with 5 mL of an isopentane solution containing 1 mg of triacontanal. **g** Sprayed with 5 mL of isopentane. **h** Sprayed with 5 mL of an isopentane solution containing 1 mg of *n*-nonacosane. All images were acquired at 2,000× magnification; scale bars = 10 μm

On the abaxial side of intact leaves of Kinkei, crystalline structures of epicuticular wax such as pectinate platelets and rodlets were highly developed (Fig. 5a). When 5 mL of isopentane was sprayed onto the intact surface of Kinkei leaves, the epicuticular wax structures changed to broad rodlets, likely due to dissolution (Fig. 5b). Spraying a solution containing 10 mg of nC29 produced large wax crusts and membrane structures that appeared to cover the natural epicuticular wax (Fig. 5c).

In contrast, intact YR leaves exhibited sparsely distributed, granular epicuticular wax on the abaxial surface, with the underlying epicuticular wax layer exposed (Fig. 5d). Mechanical wax removal eliminated the granular crystalline structures, exposing smooth surfaces beneath the epicuticular wax layer (Fig. 5e). When a solution containing 1 mg of C30al was sprayed onto the wax-removed YR leaves, membranous wax crystals formed partially, but most of the surface remained smooth (Fig. 5f).

Spraying 5 mL of isopentane onto intact YR leaves caused little change in the granular epicuticular wax crystals (Fig. 5g). In contrast, application of a solution containing 1 mg of nC29 to intact YR leaves resulted in the formation of large wax crusts and membranous structures that completely covered the treated surface (Fig. 5h).

## Discussion

The two cabbage cultivars used in this study—the waxy ‘Kinkei 201’ and the glossy ‘YR Yuryoku’—differed markedly in both epicuticular wax composition and surface morphology. Kinkei exhibited a dense and complex crystalline wax structure associated with high levels of C29-derived wax constituents, whereas YR possessed a simpler and more sparsely distributed wax layer dominated by C30al. Such differences are consistent with previous reports describing structural and compositional variation in epicuticular wax among waxy and glossy Brassicaceae cultivars (Laila et al. 2017; Macey and Barber 1970). Because wax chemistry is closely linked to wax crystal morphology (Ensikat et al. 2006), the contrasting surface structures observed between the two cabbage cultivars are likely attributable to their distinct wax compositions.

In two-choice bioassays, *P*. *xylostella* females preferentially oviposited on the glossy cultivar YR rather than on the waxy cultivar Kinkei. This finding is consistent with the results of a previous study (Justus et al. 2000). Cabbage cultivars are known to differ in a variety of traits that may affect oviposition behavior, including GLS composition, volatile profiles, leaf color, and physical leaf characteristics (Kabouw et al. 2009; Statilko et al. 2024; Wei et al. 2021). Therefore, the difference in oviposition observed between Kinkei and YR cannot be attributed solely to differences in epicuticular wax composition or crystal structure. The comparison between intact cultivars is thus best interpreted as providing ecological context rather than direct evidence of causal effects of wax traits on oviposition. To more directly evaluate the role of epicuticular wax, we compared female oviposition between surfaces with experimentally modified wax profiles on different areas of the same leaf.

Artificial removal of epicuticular wax increased oviposition on Kinkei leaves but had little effect on YR leaves. The most plausible explanation is that the dense crystalline wax structures characteristic of Kinkei provide a stronger deterrent stimulus than the relatively sparse epicuticular wax present on YR. These results support previous suggestions that leaf surface texture influences oviposition site acceptance in diamondback moths (Uematsu and Sakanoshita 1989; Uematsu and Shii 1994). Together, our findings suggest that females preferentially oviposit on smoother leaf surfaces with reduced surface roughness.

Application of nC29 restored oviposition deterrence on wax-removed Kinkei leaves and suppressed oviposition on intact YR leaves when sufficient surface coverage was achieved. However, that of C30al had little effect. Together with the SEM observations, these findings indicate that changes in leaf surface structure induced by nC29 application are closely associated with oviposition deterrence. Interestingly, when 10 mg of nC29 was applied to the intact leaf surfaces of Kinkei, females laid significantly more eggs on the treated surface than on the opposing surface, despite the extremely low mean total number of eggs per trial. This result suggests that leaf surfaces containing high amounts of nC29 are not uniformly deterrent to oviposition. One possible explanation is that application of a large amount of nC29 altered or masked the native wax architecture of Kinkei leaves, thereby changing the physical cues perceived by females. Taken together, these findings suggest that oviposition inhibition is associated with the formation of dense nC29-associated wax structures rather than simply with the presence of epicuticular wax itself, indicating that wax crystal morphology may be as important as wax chemical composition in determining oviposition behavior. However, because application of nC29 simultaneously modified both wax composition and wax crystal morphology, the relative contributions of chemical and structural cues could not be determined in the present study. In addition, the potential roles of minor wax constituents, such as 15-nonacosanone and 15-nonacosanol, were not evaluated. Future studies should investigate their individual and combined effects on oviposition deterrence and wax structure formation.

Oviposition preference in *P. xylostella* varies considerably among Brassicaceae species and cultivars (Newman et al. 2016) and has traditionally been attributed primarily to differences in GLS chemistry (Badenes-Pérez et al. 2020). Our results suggest that variation in epicuticular wax traits may represent an additional factor influencing host selection. The lower abundance of nC29-asscoiated wax structures, their simpler morphology, and the incomplete coverage of the leaf surface by epicuticular wax crystals in YR may provide tactile, and possibly chemical, cues that encourage oviposition by females. These findings indicate that variation in epicuticular wax traits can contribute substantially to oviposition preference in *P. xylostella*.

Several limitations of the experimental system should nevertheless be acknowledged. One such limitation is that females often deposited eggs on the vial opening and adjacent leaf areas. Although eggs deposited within 1 cm of the vial opening were excluded from the primary analyses to reduce positional effects associated with the experimental apparatus, reanalysis including these eggs produced qualitatively similar results (Supplementary Tables S5 and S6). Even so, the possibility that features of the experimental apparatus or substrate characteristics other than leaf wax properties influenced oviposition behavior cannot be completely excluded.

Epicuticular wax is known to function as a physical barrier against herbivorous insects by reducing attachment and locomotion on plant surfaces (Borodich et al. 2024; Eigenbrode 2004; Gorb and Gorb 2017). Previous studies have suggested that female diamondback moths have trouble maintaining stable positions on heavily waxed leaves (Zhu et al. 2022). Our findings provide further support for this hypothesis and indicate that dense wax crystal structures may reduce host acceptance during oviposition. In addition, accumulating evidence suggests that leaf and floral surface waxes contribute to the regulation of short-range attraction, host acceptance, and oviposition behavior in some herbivorous insect species (Debnath et al. 2024; Mitra et al. 2017; Mobarak et al. 2020, 2022; Udayagiri and Mason 1997). Because epicuticular wax also affect egg retention and larval movement on the leaf surface (Eigenbrode and Shelton 1990; Rahman et al. 2023; Voigt et al. 2025), it can influence insect behavior and performance throughout multiple life stages. Consequently, these findings demonstrate that epicuticular wax serves multiple ecological functions and should be viewed not merely as a defensive trait, but as a multifunctional interface mediating interactions between plants and herbivorous insects.

## Conclusion

Although host recognition and oviposition behavior in *P. xylostella* have traditionally been attributed primarily to GLSs and their derivatives (Badenes-Pérez et al. 2020), our results demonstrate that both epicuticular wax composition and wax crystal morphology contribute to oviposition preference in *P. xylostella*. In particular, oviposition deterrence was associated with the formation of dense nC29-associated wax structures rather than simply with the presence of epicuticular wax itself. However, because application of nC29 simultaneously modified both wax composition and wax crystal morphology, the relative contributions of chemical and structural cues remain unresolved. Future research should therefore disentangle the relative contributions of these cues and evaluate their consequences for female oviposition, larval performance, and offspring fitness under more realistic conditions. Such studies may facilitate the identification of wax-related traits useful for breeding Brassicaceae cultivars with enhanced resistance to *P*. *xylostella* and may contribute to the development of sustainable pest-management strategies that exploit naturally occurring plant resistance traits. 

## Supplementary Information

Below is the link to the electronic supplementary material.

Supplementary Material 1

Supplementary Material 2

Supplementary Material 3

Supplementary Material 4

Supplementary Material 5

## Data Availability

The data supporting the findings of this study are available from the corresponding author upon reasonable request.
