# Supplementary material for: Epicuticular Wax Composition and Wax Crystal Microstructure Shape Oviposition Preference in the Diamondback Moth, Plutella xylostella, on Cabbage
Source: J Chem Ecol. 2026 Nov 3;52(6):101. doi: 10.1007/s10886-026-01778-4 (PMC13727868; doi:10.1007/s10886-026-01778-4)
Supplement: Supplementary file 1 [file 10886_2026_1778_MOESM1_ESM.docx]

**Online Resource 1**

Epicuticular wax composition and wax crystal microstructure shape oviposition preference in the diamondback moth, *Plutella* *xylostella*, on cabbage

Kodai Inoue^1^, Manaka Hata^2^, Hisashi Ômura^1,3^

^1^ Graduate School of Integrated Sciences for Life, Hiroshima University

^2^ School of Applied Biological Science, Hiroshima University

^3^ Seto Inland Sea Carbon-neutral Research Center, Hiroshima University

**Quantification of wax remaining on cabbage leaves after mechanical wax removal**

Second to fourth leaves from the top of 7-week-old cabbage seedlings of cv. YR Yuryoku were detached at the base of the petiole. Epicuticular wax was mechanically removed from either the right or left half of each leaf, defined by the midvein, by gently rubbing both the adaxial and abaxial surfaces with clean cotton balls. The opposite half of the leaf was left untreated, retaining its native epicuticular wax. Each half-leaf sample was immersed in 40 mL of dichloromethane for 30 s to extract intact wax (untreated half) or residual wax (treated half). Thus, two samples were obtained from each leaf. This procedure was repeated using three independent seedlings. After extraction, the leaves were air-dried to allow solvent evaporation, mounted between transparent glass plates, and photographed using a digital camera. The area of each half leaf was determined using ImageJ 1.54d software.

The extracts were immediately subjected to gas chromatography-electron ionization mass spectrometry (GC-EIMS). Prior to analysis, an 1 mL aliquot of each sample was concentrated to 200 μL under a gentle nitrogen stream at 60 °C. GC-EIMS analysis was performed at an EI voltage of 70 eV using a QP5000 mass spectrometer coupled to a GC-17A gas chromatograph (Shimadzu, Kyoto, Japan) equipped with a Supelco Equity-1 capillary column (15 m × 0.25 mm ID, 0.25 μm film thickness: Bellefonte, PA, USA). An 1 μL aliquot of the concentrated sample was injected in splitless mode at an injector temperature of 280 °C with the split opened 30 s after injection. The oven temperature was programmed from 50 °C (held for 2 min) to 280 °C (held for 10 min) at a rate of 10 °C/min. The amount of *n*-nonacosane (nC29)was quantified with a calibration curve prepared with an authentic standard, whereas triacontanal (C30al) was semi-quantified based on its peak area relative to that of nC29. The compound contents were subsequently calculated for both treated and untreated half-leaf surfaces.

**Results**

The intact half leaves contained 1.16 μg/cm^2^ of nC29 and 4.74 μg/cm^2^ of C30al, whereas the wax-removed halves contained 0.92 μg/cm^2^ of nC29 and 4.10 μg/cm^2^ of C30al (Table 1; Fig. 1). The reduction rates of following wax removal were 20% for nC29 and 10% for C30al.

**Table 1** Comparison of wax contents between the intact half and wax-removed half leaves of cabbage cv. YR Yuryoku

**Fig. 1** Typical total ion chromatograms of chloroform extracts from the intact half (**a**) and wax-removed half (**b**) leaves of cabbage cv. YR Yuryoku. The vertical axes of the traces are at the same scale. Peaks 1 and 2 denote *n*-nonacosane and triacontanal.
