# Supplementary material for: Epicuticular Wax Composition and Wax Crystal Microstructure Shape Oviposition Preference in the Diamondback Moth, Plutella xylostella, on Cabbage
Source: J Chem Ecol. 2026 Nov 3;52(6):101. doi: 10.1007/s10886-026-01778-4 (PMC13727868; doi:10.1007/s10886-026-01778-4)
Supplement: Supplementary file 2 [file 10886_2026_1778_MOESM2_ESM.docx]

**Online Resource 2**

Epicuticular wax composition and wax crystal microstructure shape oviposition preference in the diamondback moth, *Plutella* *xylostella*, on cabbage

Kodai Inoue^1^, Manaka Hata^2^, Hisashi Ômura^1,3^

^1^ Graduate School of Integrated Sciences for Life, Hiroshima University

^2^ School of Applied Biological Science, Hiroshima University

^3^ Seto Inland Sea Carbon-neutral Research Center, Hiroshima University

**Quantification of triacontanal sprayed on wax-removed cabbage leaf surface**

Second to fourth leaves from of the top of 7-week-old cabbage seedlings of cv. YR Yuryoku were detached at the base of the petiole. Epicuticular wax was mechanically removed from both the adaxial and abaxial surfaces of the detached leaves by gently rubbing the leaf surfaces with clean cotton balls. The wax-removed half of each leaf, defined by the midvein, was covered on both sides with aluminum foil, while the exposed opposite half was sprayed with 5 mL of an isopentane–chloroform mixed solvent using a hobby airbrush. The aluminum foil was then removed. Subsequently, the previously solvent-treated half leaf was covered with aluminum foil on both sides, while the exposed opposite half was sprayed with 1 mg of triacontanal (C30al) standard dissolved in 5 mL of the mixed solvent. After removing the aluminum foil, each half of the treated leaf was individually immersed in 40 mL of chloroform for 30 s to extract surface wax. After extraction, the leaves were air-dried, mounted between transparent glass plates, and photographed with a digital camera. The area of each half leaf was determined using ImageJ 1.54d software. This sampling procedure, in which two extracts were obtained from a single treated leaf, was repeated three times using leaves collected from different seedlings.

The extracts were immediately subjected to gas chromatography-electron ionization mass spectrometry (GC-EIMS). Prior to analysis, an 1 mL aliquot of each sample was concentrated to 200 μL under a gentle nitrogen stream at 60 °C. GC-EIMS analysis was performed at an EI voltage of 70 eV using a QP5000 mass spectrometer coupled to a GC-17A gas chromatograph (Shimadzu, Kyoto, Japan) equipped with a Supelco Equity-1 capillary column (15 m × 0.25 mm ID, 0.25 μm film thickness: Bellefonte, PA, USA). An 1 μL aliquot of the concentrated sample was injected in splitless mode at an injector temperature of 280 °C with the split opened 30 s after injection. The oven temperature was programmed from 50 °C (held for 2 min) to 280 °C (held for 10 min) at a rate of 10 °C/min. The amount of C30al was semi-quantified based on its peak area relative to that of *n*-nonacosane, which was quantified using a calibration curve prepared with an authentic standard. The average amount of C30al per unit leaf area was subsequently calculated for each half leaf.

**Result**

The average amount of C30al per unit leaf area was 5.5 μg/cm^2^ for the half leaf subjected to wax removal followed by spraying with the mixed solvent, and 12.3 μg/cm^2^ for the half leaf subjected to wax removal followed by spraying 1 mg of C30al dissolved in the mixed solvent, respectively (Table 1; Fig. 1). These results indicate that spraying 1 mg of C30al increased the amount of C30al remaining on the leaf surface after wax removal by approximately twofold.

**Table 1** Quantification of triacontanal deposition on the wax-removed leaf surface of cabbage cv. YR Yuryoku after application of 1 mg triacontanal standard

**Fig. 1** Typical total ion chromatograms of chloroform extract from leaves of cabbage cv. YR Yuryoku after the following treatments: (**A**) wax removal and spraying 5 mL of isopentane-chloroform mixed solvent and (**B**) wax removal and spraying 1 mg of triacontanal dissolved in 5 mL of the mixed solvent. The total ion intensities of two traces are displayed at the same scale. Peaks with an asterisk denote triacontanal.
