# Supplementary material for: Epicuticular Wax Composition and Wax Crystal Microstructure Shape Oviposition Preference in the Diamondback Moth, Plutella xylostella, on Cabbage
Source: J Chem Ecol. 2026 Nov 3;52(6):101. doi: 10.1007/s10886-026-01778-4 (PMC13727868; doi:10.1007/s10886-026-01778-4)
Supplement: Supplementary file 3 [file 10886_2026_1778_MOESM3_ESM.docx]

**Online Resource 3**

Epicuticular wax composition and wax crystal microstructure shape oviposition preference in the diamondback moth, *Plutella* *xylostella*, on cabbage

Kodai Inoue^1^, Manaka Hata^2^, Hisashi Ômura^1,3^

^1^ Graduate School of Integrated Sciences for Life, Hiroshima University

^2^ School of Applied Biological Science, Hiroshima University

^3^ Seto Inland Sea Carbon-neutral Research Center, Hiroshima University

**Quantification of *n*-nonacosane sprayed on intact cabbage leaf surface**

Second to fourth leaves from the top of 7-week-old seedlings of cabbage cv. YR Yuryoku were detached at the base of the petiole. Half of each detached leaf, defined by the midvein, was covered on both the adaxial and abaxial surfaces with aluminum foil, whereas the exposed opposite half was sprayed on both surfaces with 5 mL of isopentane using a hobby airbrush. The aluminum foil was then removed. Subsequently, the previously solvent-treated half leaf was covered with aluminum foil on both surfaces, while the exposed opposite half was sprayed on both surfaces with 0.1 mg or 1 mg of *n*-nonacosane (nC29) standard dissolved in 5 mL of isopentane. After removing the aluminum foil, each half of the treated leaf was individually immersed in 40 mL of chloroform for 30 s to extract surface wax. After extraction, the leaves were air-dried, mounted between transparent glass plates, and photographed with a digital camera. The area of each half leaf was determined using ImageJ 1.54d software. This sampling procedure, in which two extracts were obtained from a single treated leaf, was repeated three times using leaves collected from different seedlings.

The extracts were immediately subjected to gas chromatography-electron ionization mass spectrometry (GC-EIMS). Prior to analysis, an 1 mL aliquot of each sample was concentrated to 200 μL under a gentle nitrogen stream at 60 °C. GC-EIMS analysis was performed at an EI voltage of 70 eV using a QP5000 mass spectrometer coupled to a GC-17A gas chromatograph (Shimadzu, Kyoto, Japan) equipped with a Supelco Equity-1 capillary column (15 m × 0.25 mm ID, 0.25 μm film thickness: Bellefonte, PA, USA). An 1 μL aliquot of the concentrated sample was injected in splitless mode at an injector temperature of 280 °C with the split opened 30 s after injection. The oven temperature was programmed from 50 °C (held for 2 min) to 280 °C (held for 10 min) at a rate of 10 °C/min. The amount of nC29 was quantified with a calibration curve prepared with an authentic standard, whereas triacontanal (C30al) was semi-quantified based on its peak area relative to that of nC29. The average amounts of nC29 and C30al per unit leaf area was subsequently calculated for each half leaf.

**Result**

The half leaf sprayed with 5 mL of isopentane contained 3.3 μg/cm^2^ of nC29 and 7.0 μg/cm^2^ of C30al, whereas the half sprayed with 0.1 mg of nC29 dissolved in the solvent had 5.0 μg/cm^2^ of nC29 and 6.8 μg/cm^2^ of C30al (Table 1, Fig. 1). Therefore, spraying 0.1 mg of nC29 resulted in only a small increase in the amount of nC29 on the leaf surface. In contrast, spraying 1 mg of nC29 increased the amount of nC29 on the leaf surface approximately fourfold, from 3.6 μg/cm^2^ to 18.0 μg/cm^2^ (Table 2, Fig. 2). The ratio of nC29 to C30al also increased from 0.65 to 4.4 (Table 2), indicating that this treatment altered the composition of leaf surface wax.

**Table 1** Quantification of *n*-nonacosane and triacontanal deposition on the intact leaf surface of cabbage cv. YR Yuryoku after application of 0.1 mg *n*-nonacosane standard

**Fig. 1** Typical total ion chromatograms of chloroform extract from leaves of cabbage cv. YR Yuryoku after the following treatments: (**a**) spraying 5 mL isopentane-chloroform mixed solvent and (**b**) spraying 0.1 mg of *n*-nonacosane in 5 mL of the mixed solvent. The total ion intensities of two traces are displayed at the same scale. Peaks with an asterisk denote *n*-nonacosane.

**Table 2** Quantification of *n*-nonacosane and triacontanal on the intact leaf surface of cabbage cv. YR Yuryoku after application of 1 mg *n*-nonacosane standard

**Fig. 2** Typical total ion chromatograms of chloroform extract from leaves of cabbage cv. YR Yuryoku after the following treatments: (**a**) spraying 5 mL of isopentane-chloroform mixed solvent and (**b**) spraying 1 mg of *n*-nonacosane in 5 mL of the mixed solvent. The total ion intensities of two traces are displayed at the same scale. Peaks with an asterisk denote *n*-nonacosane.
