# Supplementary material for: Epicuticular Wax Composition and Wax Crystal Microstructure Shape Oviposition Preference in the Diamondback Moth, Plutella xylostella, on Cabbage
Source: J Chem Ecol. 2026 Nov 3;52(6):101. doi: 10.1007/s10886-026-01778-4 (PMC13727868; doi:10.1007/s10886-026-01778-4)
Supplement: Supplementary file 4 [file 10886_2026_1778_MOESM4_ESM.docx]

**Online Resource 4**

Epicuticular wax composition and wax crystal microstructure shape oviposition preference in the diamondback moth, *Plutella* *xylostella*, on cabbage

Kodai Inoue^1^, Manaka Hata^2^, Hisashi Ômura^1,3^

^1^ Graduate School of Integrated Sciences for Life, Hiroshima University

^2^ School of Applied Biological Science, Hiroshima University

^3^ Seto Inland Sea Carbon-neutral Research Center, Hiroshima University

**Effects of spraying with isopentane on oviposition by *Plutella* *xylostella***

Two-choice bioassays were performed to examine whether foliar spraying with isopentane affects female oviposition of *Plutella* *xylostella* on cabbage cultivars Kinkei 201 and YR Yuryoku. Second to fourth leaves from the top of cabbage seedlings older than 7 weeks were detached at the base of petiole. Half of each detached leaf, defined by the midvein, was covered on both the adaxial and abaxial surfaces with aluminum foil, whereas the exposed opposite half was sprayed on both sides with 5 mL of isopentane using a hobby airbrush. The aluminum foil was then removed, and the treated half was allowed to air-dry. The prepared leaf was placed vertically in a 5 mL glass vial (8 mm inner diameter at the top, 45 mm height) containing water, and the vial was positioned at the center of the floor of a box-shaped test chamber (34 cm × 26 cm × 34 cm). Eight mated females, 3 days after emergence, were randomly selected from the stock culture and released into the chamber. The insects were allowed to fly and oviposit freely for 3 h in the dark at 25 °C. To minimize positional bias between treated and intact halves, the leaf orientation was reversed 1.5 h after the start of the bioassay. The number of eggs laid on each half-leaf surface, at least 1 cm above the top of the vial, was recorded. Each experiment was repeated at least 10 times using different individuals, and a total of 15 replicates were conducted.

**Result**

In both Kinkei 201 and YR Yuryoku, foliar spraying with 5 mL of isopentane had no significant effect on oviposition of *P*. *xylostella* (Fig. 1; quasibinomial GLM: *P* = 0.304 for Kinkei 201 and *P* = 0.277 for YR Yuryoku). The mean numbers of eggs laid on the untreated and treated leaf halves were 5.6 and 3.7, respectively, in Kinkei 201 (Table 1) and 21.2 and 17.5, respectively, in YR Yuryoku (Table 2). Notably, females laid an average of approximately 26 eggs per trial in the grooves around the threaded neck of the glass vial, exceeding the number of eggs laid on the leaves of Kinkei 201. This observation indicates that *P*. *xylostella* females preferentially oviposit in depressions, even on artificial substrates (Fig. 2). Furthermore, in trials 4 and 14 of the Kinkei 201 bioassay, many eggs were deposited on leaf areas located within 1 cm of the vial opening. Direct observations showed that females frequently hung upside down from the threaded portion of the vial opening and deposited eggs on adjacent leaf surfaces that could be reached by the abdomen. Therefore, eggs deposited on the vial itself or within 1 cm of the vial opening were excluded from subsequent analyses.

**Fig. 1** Oviposition preference of *Plutella* *xylostella* on a single cabbage leaf of (**a**) cv. Kinkei 201 (conventional) and (**b**) cv. YR Yuryoku (waxless), with different treatments applied to the left and right halves defined by the midvein: intact half vs intact half sprayed with 5 mL of isopentane (solvent). Number of trials are shown in parentheses. Solvent treatments did not significantly affect female oviposition (Quasibinomial GLM: *P* = 0.304 for Kinkei 201 and *P* = 0.277 for YR Yuryoku).

**Fig. 2** *Plutella* *xylostella* females ovipositing on a test leaf and at the screw neck of a vial

**Table 1** Raw data from bioassays for Kinkei 201

|  | Number of eggs laid | |  |  |
| --- | --- | --- | --- | --- |
| No of trials | Intact (untreated) leaf surface | treated leaf surface (sprayed with isopentane) | Screw neck of vial | Leaf surface within 1 cm of the top of the vial |
| 1 | 1 | 2 | 52 | 0 |
| 2 | 3 | 2 | 4 | 0 |
| 3 | 13 | 13 | 14 | 0 |
| 4 | 0 | 3 | 53 | 11 |
| 5 | 0 | 0 | 64 | 2 |
| 6 | 0 | 2 | 0 | 0 |
| 7 | 0 | 0 | 18 | 0 |
| 8 | 0 | 0 | 18 | 0 |
| 9 | 0 | 0 | 65 | 0 |
| 10 | 0 | 5 | 35 | 0 |
| 11 | 6 | 9 | 1 | 0 |
| 12 | 4 | 2 | 0 | 4 |
| 13 | 2 | 10 | 9 | 5 |
| 14 | 4 | 0 | 5 | 18 |
| 15 | 51 | 7 | 43 | 2 |
| Mean | 5.6 | 3.7 | 25.4 | 2.8 |
| S.D. | 12.6 | 4.1 | 23.4 | 5.0 |

**Table 2** Raw data from bioassays for YR Yuryoku

|  | Number of eggs laid | |  |  |
| --- | --- | --- | --- | --- |
| No of trials | Intact (untreated) leaf surface | treated leaf surface (sprayed with isopentane) | Screw neck of vial | Leaf surface within 1 cm of  the top of the vial |
| 1 | 44 | 28 | 5 | 0 |
| 2 | 4 | 2 | 44 | 0 |
| 3 | 20 | 19 | 37 | 0 |
| 4 | 16 | 9 | 64 | 0 |
| 5 | 7 | 10 | 35 | 0 |
| 6 | 18 | 12 | 19 | 0 |
| 7 | 74 | 42 | 54 | 0 |
| 8 | 9 | 29 | 36 | 0 |
| 9 | 36 | 6 | 25 | 0 |
| 10 | 19 | 20 | 5 | 0 |
| 11 | 8 | 11 | 8 | 0 |
| 12 | 7 | 7 | 41 | 0 |
| 13 | 48 | 40 | 27 | 0 |
| 14 | 3 | 7 | 0 | 0 |
| 15 | 5 | 21 | 0 | 0 |
| Mean | 21.2 | 17.5 | 26.7 | 0 |
| S.D. | 19.8 | 12.0 | 19.5 | 0.0 |
