# Supplementary material for: Epicuticular Wax Composition and Wax Crystal Microstructure Shape Oviposition Preference in the Diamondback Moth, Plutella xylostella, on Cabbage
Source: J Chem Ecol. 2026 Nov 3;52(6):101. doi: 10.1007/s10886-026-01778-4 (PMC13727868; doi:10.1007/s10886-026-01778-4)
Supplement: Supplementary file 5 [file 10886_2026_1778_MOESM5_ESM.docx]

Supplementary Information

Epicuticular wax composition and wax crystal microstructure shape oviposition preference in the diamondback moth, *Plutella* *xylostella*, on cabbage

Kodai Inoue^1^, Manaka Hata^2^, Hisashi Ômura^1,3 *^

**Authors information**

**Affiliations**

1 Graduate School of Integrated Sciences for Life, Hiroshima University, Higashihiroshima 739-8528, Japan

2 School of Applied Biological Science, Hiroshima University, Higashihiroshima,

Japan, 739-8528

3 Seto Inland Sea Carbon-neutral Research Center, Hiroshima University, Higashihiroshima 739-8528, Japan

**Corresponding author**

Correspondence to Hisashi Ômura

E-mail address: homura@hiroshima-u.ac.jp

**Figure S1** Total ion chromatogram (**A**) and mass spectrum (**B**) of synthetic triacontanal used as an authentic standard. Chromatogram was obtained using a Supelco Equity-1 capillary column (15 m × 0.25 mm ID), with the oven temperature programmed from 50 °C (initial hold for 2 min) to 280 °C (final hold for 10 min) at 10 °C/min.

**Figure S2** Representative total ion chromatograms of chloroform extracts from intact leaves of cabbage cultivars Kinkei 201 (**A**) and YR Yuryoku (**B**). Chromatogram was obtained using a Supelco Equity-1 capillary column (15 m × 0.25 mm ID), with the oven temperature programmed from 50 °C (initial hold for 2 min) to 280 °C (final hold for 10 min) at 10 °C/min. Peak numbers correspond to those in Table 1.

**Table S1** Peak areas of four major peaks in chloroform extracts from intact leaves of cabbage cultivar Kinkei 201 (1 μL injection)

|  |  | Peak area |  |  |  |  |  |  |  |  |  |
| --- | --- | --- | --- | --- | --- | --- | --- | --- | --- | --- | --- |
| No. | Compound | Sample 1 | Sample 2 | Sample 3 | Sample 4 | Sample 5 | Sample 6 | Sample 7 | Sample 8 | Sample 9 | Sample 10 |
| 1 | *n*-Nonacosane | 30465826 | 31101717 | 34240384 | 101469204 | 98615049 | 99440882 | 116568364 | 105113216 | 90937476 | 115892404 |
| 2 | 15-Nonacosanone | 12712813 | 14816483 | 14695032 | 68223980 | 61005903 | 58888285 | 73275848 | 61564715 | 48153492 | 71450782 |
| 3 | 15-Nonacosanol | 8388411 | 7816579 | 8434240 | 42821944 | 34229855 | 32572365 | 39776496 | 29254918 | 19874744 | 41278647 |
| 4 | Triacontanal | 10784548 | 3990263 | 8961212 | 35918467 | 43935053 | 28033270 | 24184082 | 11584449 | 8687651 | 24775008 |

**Table S2** Peak areas of four major peaks in chloroform extracts from intact leaves of cabbage cultivar YR Yuryoku (1 μL injection)

|  |  | Peak area |  |  |  |  |  |  |  |  |  |
| --- | --- | --- | --- | --- | --- | --- | --- | --- | --- | --- | --- |
| No. | Compound | Sample 1 | Sample 2 | Sample 3 | Sample 4 | Sample 5 | Sample 6 | Sample 7 | Sample 8 | Sample 9 | Sample 10 |
| 1 | *n*-Nonacosane | 1260831 | 762266 | 979891 | 8044757 | 4223354 | 8651352 | 3399706 | 6193220 | 8766914 | 6509570 |
| 2 | 15-Nonacosanone | 509456 | 386803 | 505544 | 9188451 | 4930644 | 8842301 | 3500926 | 7085352 | 8494190 | 3614555 |
| 3 | 15-Nonacosanol | 75481 | 39302 | 35325 | 1654951 | 565164 | 1139192 | 102345 | 418498 | 877002 | 238974 |
| 4 | Triacontanal | 7486337 | 7322328 | 6706841 | 22622853 | 14530023 | 17823792 | 18112977 | 19920672 | 23258976 | 10379608 |

**Table S3** Number of eggs in each oviposition bioassay trial

**Table S4** Summary of statistical analyses of oviposition bioassay

Asterisks indicate significant differences (* for *P* < 0.05, ** for *P* < 0.01, and *** for *P* < 0.001)

**Table S5** Number of eggs laid in each oviposition bioassay trial, including eggs deposited within 1 cm of the vial opening

**Table S6** Summary of statistical analyses of oviposition bioassay, including eggs deposited within 1 cm of the vial opening

Asterisks indicate significant differences (* for *P* < 0.05, ** for *P* < 0.01, and *** for *P* < 0.001)
